# Smart waste management perspective of COVID-19 healthy personal protective materials in concrete for decorative landscape pavements and artificial rocks

**DOI:** 10.1038/s41598-023-30104-1

**Published:** 2023-02-18

**Authors:** Ahmed Abd El Aal, Mabkhoot A. Alsaiari, Ahmed E. Radwan, Amr Fenais

**Affiliations:** 1 Civil Engineering Department, Faculty of Engineering, Najran University, Najran, Kingdom of Saudi Arabia; 2 Advanced Materials and Nanotechnology Research Centre, Najran University, Najran, Kingdom of Saudi Arabia; 3 Empty Quarter Research Unit, Chemistry Department, Faculty of Science and Arts at Sharurah, Najran University, Najran, Kingdom of Saudi Arabia; 4 Faculty of Geography and Geology, Institute of Geological Sciences, Jagiellonian University, Gronostajowa 3a, 30-387 Kraków, Poland

**Keywords:** Environmental sciences, Environmental social sciences, Engineering, Materials science, Physics

## Abstract

This paper presents a new method for determining the effect of healthy personal protective material (HPPM) stripes, such as surgical masks, protective suits, and overhead and foot covers, on the durability and physicomechanical characteristics of concrete for use in architectural forms. Because of the current global epidemic caused by coronavirus (COVID-19), the use of HPPM, such as surgical masks, protective suits, and overhead and foot covers, has increased considerably. COVID-19’s second and third waves are currently affecting various countries, necessitating the use of facemasks (FM). Consequently, millions of single FM have been discharged into the wild, washing up on beaches, floating beneath the seas, and ending up in hazardous locations. The effect of stripe fibers on the physicomechanical characteristics of concrete, such as the workability, Uniaxial Compressive Strength UCS, flexural strength, impact strength, spalling resistance, abrasion resistance, sorptivity, Water absorption Sw, porosity (ηe), water penetration, permeability, and economic and eco-friendly aspects, need to be determined. With a focus on HPPM, especially single-use facemasks, this study investigated an innovative way to incorporate pandemic waste into concrete structures. Scanning electron microscope and X-ray diffraction patterns were employed to analyze the microstructures and interfacial transition zones and to identify the elemental composition. The HPPM had a pore-blocking effect, which reduced the permeability and capillary porosity. Additionally, the best concentrations of HPPM, particularly of masks, were applied by volume at 0, 1, 1.5, 2.0, and 2.5%. The use of mixed fibers from different HPPMs increased the strength and overall performance of concrete samples. The tendency of growing strength began to disappear at approximately 2%. The results of this investigation showed that the stripe content had no effect on the compressive strength. However, the stripe is critical for determining the flexural strength of concrete. The UCS increased steadily between 1 and 1.5% before falling marginally at 2.5%, which indicates that incorporating HPPM into concrete had a significant impact on the UCS of the mixture. The addition of HPPM to the mixtures considerably modified the failure mode of concrete from brittle to ductile. Water absorption in hardened concrete is reduced when HPPM stripes and fibers were added separately in low-volume fractions to the concrete mixture. The concrete containing 2% HPPM fibers had the lowest water absorption and porosity percentage. The HPPM fibers were found to act as bridges across cracks, enhancing the transfer capability of the matrices. From a technological and environmental standpoint, this study found that using HPPM fibers in the production of concrete is viable.

## Introduction

Concrete has a strong compressive strength but a tensile strength ten times that of steel. It also has a brittle property, which prevents stress transmission after cracking. It is feasible to add fibers to concrete mixes to prevent brittle failure and increase the mechanical qualities. Healthy Personal Protective Materials (HPPM) stripes are cementitious composite materials with scattered fibers, such as steel, polymer, polypropylene, carbon, and glass^1^. The protection of steel bars from corrosion and sulfate attack, as well as from water and ion infiltration through pores and cracks, is linked to improving the longevity of reinforced concrete^2^. As a result, both the insertion of fibers and the replacement of traditional reinforcement with fibers are favourable in terms of long-term development^1^. Polypropylene fiber-reinforced concrete was experimentally investigated by^3^. The compressive strength decreased marginally over the testing period after the addition of a 3% polypropylene stripe by volume, with the most significant reduction at 10%. The splitting tensile strength improved by 39%, whereas compressive strength reduced with the inclusion of a 1% polypropylene stripe by volume.

In comparison to the fiber with the smallest size, scientists found that increasing the quantity of the fiber increased compressive, tensile, and flexural strengths by 10, 14, and 58%, respectively. Furthermore, based on the findings of^4^, thin fibers may be a viable solution for reducing creep strain. The efficiency of fibers is determined not only by the metrics listed above but also by their concrete bonding strength^5^. In addition, fibers can be crimped, twisted, sinusoidal, or hooked to improve their contact surface with the matrix, and their indents can be fibrillated (the ends split during mixing). The mechanical properties of concrete mixtures are also affected by the fiber form^6^.

Xu et al.^7^ conducted similar experiments on fiber-reinforced concrete and discovered that when cellulose fiber (CTF) was used at dosages of 1.5 kg/m^3^, the concrete's Uniaxial Compressive Strength (UCS) increased to 12%; however, when polyvinyl alcohol fiber (PF) was used at dosages of approximately 4.0 kg/m^3^, the concrete’s UCS was reduced by 35%. When the dosage was increased to 2.0 kg/m^3^, CTF's splitting tensile strength declined by 23%, while PF's decreased by 55%. The splitting tensile strength of the polyolefin fibers also degraded. Additionally, the use of fiber reinforcement in concrete imposes specific mix composition constraints; therefore, it may be necessary to make changes^8^. The number, form, and slenderness of fibers affect the workability of concrete^1,9–11^. It’s a promising field of use, particularly in metropolitan locations where there are adverse environmental conditions, damage caused by environmental conditions, abrasion of surfaces, and vandalism. However, HPPM fibers are more commonly used in architectural applications. Specifically, HPPM fibers are particularly successful in reducing plastic shrinkage fractures soon after concrete is made, and they considerably improve its post-cracking behavior.

Kilmartin-Lynch et al.^12^ have presented an innovative method for integrating single-use face masks in the production of concrete. The method examined the impact of adding PPE on enhancing the mechanical characteristics of concrete by using cement and other aggregates that are frequently found in Australia, together with water reducer, and using rare lower amounts of PPE (i.e., 0.10%, 0.15%, 0.20%, and 0.25%).

Koniorczyk et al.^13^ used the recommended dosage of 1 mask per 1 L of concrete. According to their findings, adding processed masks improved compressive strength (by about 5%) and tensile strength (by about 3%).

Castellote et al.^14^ added surgical wear masks (WM) to mortars in amounts up to 5% of the cement weight. The characterization of the mechanical and microstructural aspects has been done in their work. The findings show that adding MW to cement precludes a deterioration in the material's qualities, including strength and durability behavior.

Researchers have identified a number of benefits of employing HPPM in concrete mixtures, but few studies have been conducted on enhancing the durability and engineering qualities of concrete for use in buildings. Owing to the strong effect of HPPM on concrete behavior, multiple laboratory samples containing various percentages of HPPM were made in this study by utilizing the standard mix of concrete for architectural fabrication.

The primary goal of this study was to explore whether single-use facemasks can be recycled and reused to reduce the amount of pandemic-related garbage that ends up in landfills or litters the streets during this crisis. We studied the influence of HPPM fibers on the physical and mechanical properties of concrete and how HPPM fibers might be employed in a potential sector of application, such as public spaces. This is an interdisciplinary study that involves both engineering and architecture. This revolutionary technique provides understanding of this topic by integrating various areas of knowledge and is noteworthy because no previous study has been published on the use of HPPM fibers in public settings.

## Materials and methods

### Materials

#### Cement

Type I ordinary Portland cement from Najran cement (OPC) was used in this study. The specific gravity of the cement was 3.15, and it had a Blaine fineness of 410 m^2^/kg. The Bogue phases of the cement, according to the manufacturer, were 59% C3S, 12.1% C2S, 10.6% C3A, and 10.4% C4AF. The oxides found in the cement are listed in Table 1.

**Table 1:** Cement chemical composition.

Items	SiO_2_	Al_2_O_3_	Fe_2_O_3_	CaO	SO_3_	MgO	K_2_O	Insoluble	LOI
wt%	19.73	6.2	3.44	63.78	2.23	0.96	1.02	0.93	1.51

#### Aggregates

To cast the concrete samples that met ASTM C33/C33M-18, the fine aggregate was gathered from natural sand with a maximum size of 4.75 mm, and the coarse aggregate was natural crushed stone with a maximum size of 20 mm. The physical parameters of the aggregates are presented in Table 2. Coarse sand was employed as a fine aggregate in the concrete samples, and crushed stone chips complying with ASTM C33 were used as coarse aggregates. Table 2 lists the physical features of these aggregates.

**Table 2:** Material properties.

Material	Fineness modulus	Specific gravity	Absorption (%)	Dry rodded unit weight (kg/m^3^)
Coarse aggregate	–	2.81	0.46	1555
Fine aggregate	2.43	2.63	1.65	1596
(HPPM)	–	–	8.8	–

#### Water

Concrete mixtures and curing were performed using potable tap water. The ASTM C1602/C1602M criterion was satisfied for the properties of water.

#### Characteristic of polypropylene stripes (HPPM)

**Figure 1:**
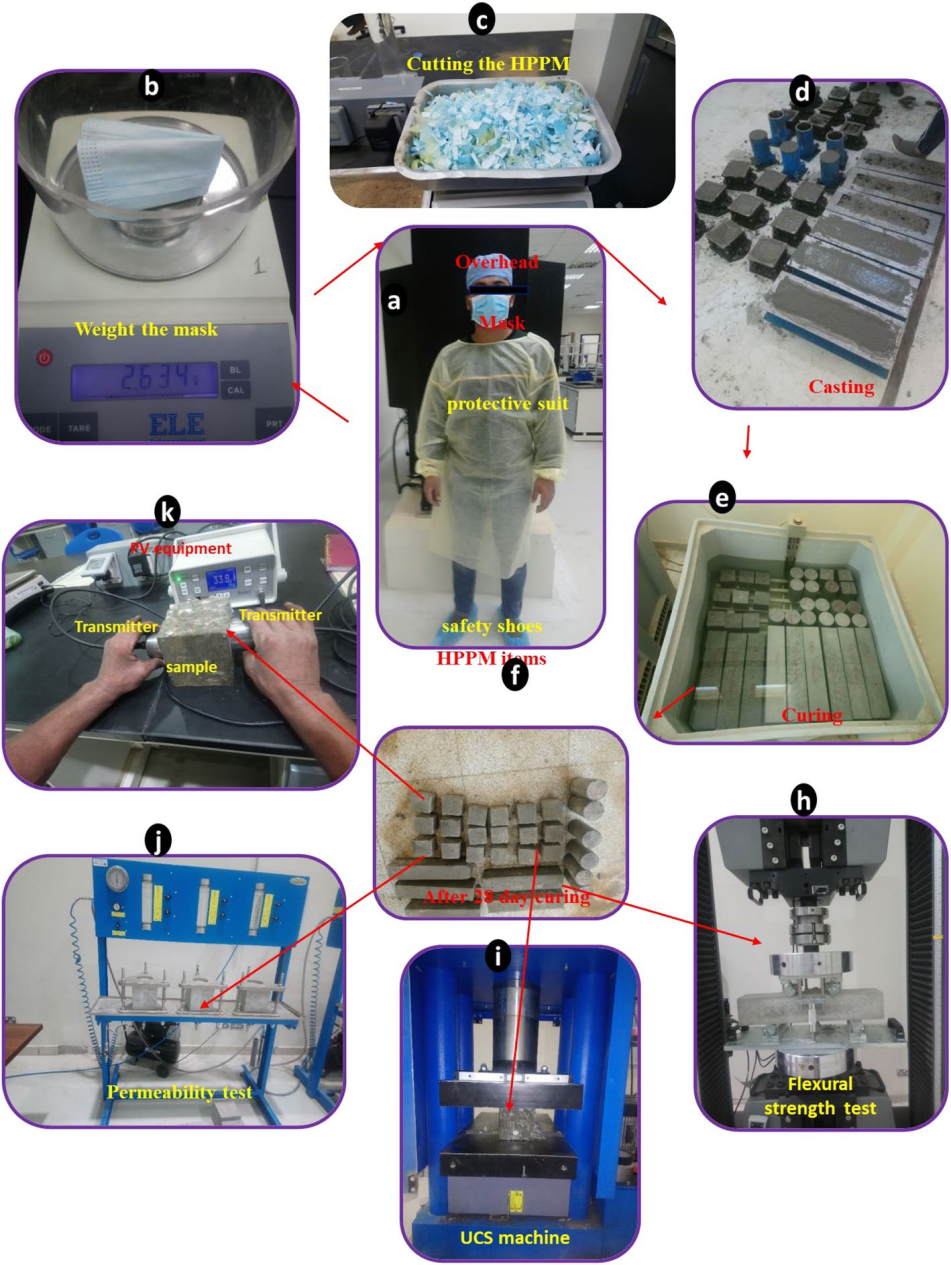
Experiment protocols, casting, and curing of the concrete with HPPM.

The polypropylene stripes (HPPM) used in this study are commercially available, such as polypropylene stripes from surgical masks, protective suits, and overhead and foot covers, as indicated in Fig. 1. The collected polypropylene stripes from surgical masks, protective suits, and overhead and foot covers have the same properties^12,13,15^. Since the polypropylene stripes have the same properties so the different proportions of these HPPMs will not affect the results. Polypropylene fibers were mixed into concrete at a rate of 2.5% of the total volume.

## Experimental design

The samples mixed with cut-up HPPM were analyzed using six concrete combinations in proportions of 0% (control mix), 0.5%, 1%, 1.5%, 2%, and 2.5% by volume of concrete (three samples for each mix). This choice is in line with prior studies by^3,7,16^. Najran cement with a specific gravity of 3.15 and a bulk density of 1250–1650 kg/m^3^ was utilized throughout the experiments, as well as coarse aggregates with a nominal size of 20 mm and fine aggregates with a specific gravity of 2.63, which were oven-dried for 48 h at 110 °C to remove excess moisture. Table 1 lists the cement parameters used. The physical parameters of the HPPM and fine and coarse aggregates are listed in Table 2. New and unused HPPM were utilized in this study to limit community transmission and infection risk from the coronavirus. The HPPM was cut into small pieces with a length of 1 cm and a width of 1 cm (Fig. 1).

### Concrete mix proportions

Table 3 shows the mix design used to cast the samples and the varied amounts of HPPM. CM0 denotes a control mix containing no surgical masks, whereas CM25 denotes concrete with a 2.5% by volume concentration.

**Table 3:** Mix proportions of concrete.

Weights by kg	Percentage %					
	0	0.5	1	1.5	2.0	2.5
Water	217	217	217	217	217	217
Cement	400	400	400	400	400	400
Coarse Agg	1054	1054	1054	1054	1054	1054
Fine Agg	666	666	666	666	666	666
HPPM	0	11.68	23.36	35.04	46.72	58.4

In a reference mixer, the concrete samples were prepared according to ASTM C192M with a water/cement ratio of 0.50. No additional materials or chemical admixtures were used in this study. HPPM fibers were employed in the proportions of 0.5, 1, 1.5, 2.0, and 2.5% as an extra percentage. The concrete was mixed and placed into various molds (cubes, cylinders, and prisms) for 24 h before being demolded and cured in clean drinkable tap water. The concrete samples were cured for 28 d at room temperature from 21 to 24 °C (Fig. 1).

### Casting and curing

All dry materials were weighed and then mixed in a concrete mixer for 3 min to mix. After 3 min of mixing with water, the dry materials were gently added and combined for another 3 min. After detachment from the mixer, concrete was poured into cylindrical molds. To avoid concrete adhesion, the inner surfaces of the assembled molds were thinly coated with mold oil. To allow the concrete to settle, cylindrical molds were filled with concrete and placed on a vibrating table for 20 s. The molds were filled with concrete after the first 20 s and then vibrated for another 20 s to ensure the absence of voids. The new concrete surface was finished by using a smooth steel trowel. The specimens were taken from the molds after 24 h and immediately submerged in clean fresh water for 28 d for the strength test. The procedure was performed for each concrete batch. Previous research ^3,16,17^ has employed identical casting procedures (1).

### Experimental details

The compressive, split tensile, and flexural strength tests were conducted using^18–20^ respectively. The compressive strength test was performed on 100 × 100 × 100 mm^3^ cube specimens (The results corrected to 15 * 15 cubes according to ASTM C39/C39M-21^18^), while the split test was performed on samples that were 100 mm in diameter and a height of 200 mm and had a flexural strength of 100 mm diameter, 200 mm height, prism specimens. The cube specimens were weighed in water immediately after they were removed from the water basin in the saturated surface dry state and in the dry state to determine the sample densities. The compression testing apparatus had a force of 2000 kg per square meter. Three samples from each mix configuration were inspected for defects before they were subjected to a force of 157 kN/min. To assess the homogeneity and structural integrity of the manufactured HPPM concrete, non-distractive pulse velocity (PV) testing was performed on compression samples in accordance with^21^. This test can be used to assess the consistency and uniformity of the concrete samples and to assess the cracks and voids that are not visible on the surface. To evaluate the effective porosity, two to three representative specimens (with individual masses > 50 g) from a sample were immersed in water in a desiccator (e). A vacuum pressure of > 800 Pa (required by^22^) was maintained within the desiccator for at least 2 h to saturate the specimens. The dry (M_dry_) and saturated (M_sat_) masses of a specimen and a saturated specimen suspended in water (M_sus_) were calculated. To calculate the effective porosity (e) and dry density (dry), the following equations were used:

1 $$\eta {\text{e }} = {\text{ M}}_{{{\text{sat}}}} {-}{\text{M}}_{{{\text{dry}}}} /{\text{M}}_{{{\text{sat}}}} {-}{\text{M}}_{{{\text{SSD}}}}$$

where ηe = Porosity %, M_sat_: saturated mass, M_dry_: dry mass, M_SSD_: saturated surface dry mass.

After 28 d of curing, the concrete samples were removed from the curing tank and allowed to dry. The tops of the cylindrical samples were ground back after air-drying to produce a smooth contact surface with the compression and testing gear, as per ASTM C31/C31M-21. The loading rate was 0.34 MPa/s^18^.

A Hitachi U8040 scanning electron microscope was used for the scanning electron microscopy (SEM) study. The liquid permeabilities of the hardened specimens were determined according to^23^. A 150-mm cube specimen was used for the permeability test. Before we measured the permeability, the specimen was cured for 28 d. Figure 1 depicts the experimental setup for all the tests.

## Results

HPPM stripes (polypropylene) have been utilized in concrete as crack-resistant improvement materials. The HPPM was constructed from plastic polypropylene fibers according to the manufacturer’s instructions. It also exhibits high chemical stability and strength. By volume, the fiber content ranged from 0.5 to 2.5%. HPPMs were chopped into small pieces that were 1 cm in length and 1 cm in width. The physical parameters of single-use HPPM are listed in Table 4.

**Table 4:** Physical properties of healthy personal protective materials (HPPM) from Shabrian et al.^24^.

Physical properties	SHM	Standard
Specific gravity	0.91	^25^
Melting point (°C)	160	^26^
Water absorption 24 h (%)	8.8	^27^
Tensile strength (MPa)	3.65	^28^
Tensile strength at break (MPa)	3.97	^28^
Elongation at break (%)	118.9	^28^
Rupture force (N)	19.46	^28^
Aspect ratio	24	–

### XRD and SEM of the HPPM stripes

**Figure 2:**
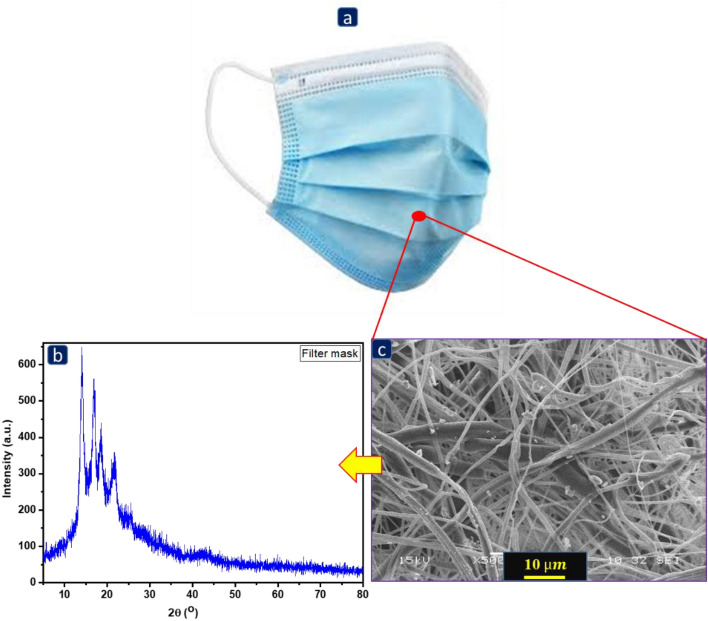
(**a**) Image of single-use facemask, (**b**) XRD image of FM fibers, and (**c**) appearance of facemask layers under scanning electron microscopy at 1000× .

Figure 2a,b show the facemask and HPPM fiber integrated X-ray diffraction patterns. The diffraction peaks of all fibers were obtained between 10 and 30 degrees, as shown in Fig. 2b. The peaks obtained at approximately 14°, 17°, 18.6°, 21–22°, and 28° are similar to the peaks generated by polypropylene. Any microstructural changes in HPPM were observed using SEM. The HPPM layer (polypropylene) was sliced into a 10 mm × 10 mm size and examined using a SEM (Hitachi, TM3000) at 1000× magnification. Figure 2c shows the structural changes in the polypropylene fibers, such as melting, distortion, tangling, and cracking.

### XRD results of concrete containing HPPM fibers

**Figure 3:**
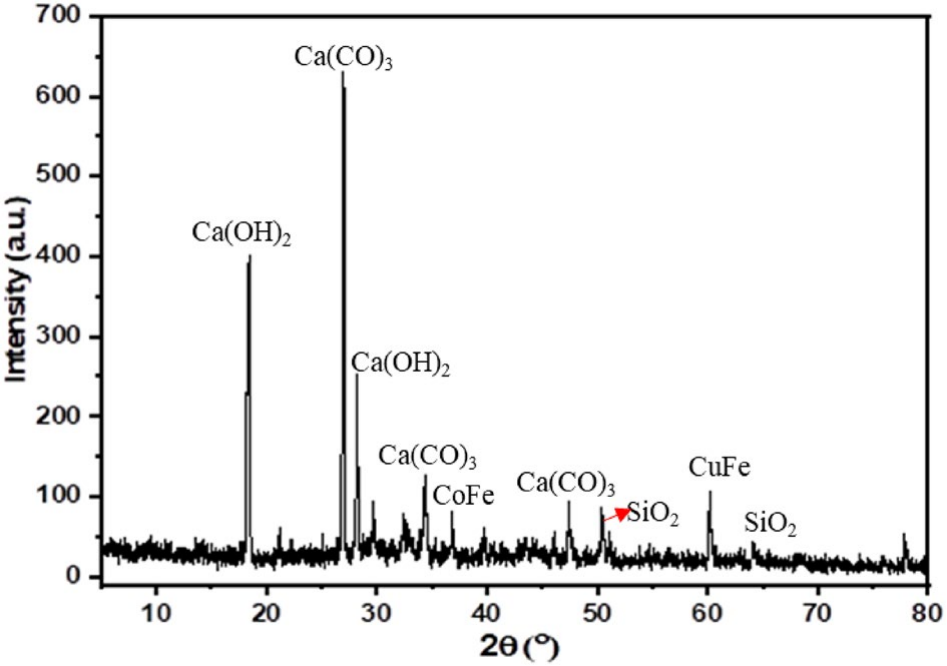
XRD analysis of concrete with 2% HPPM after 28 days.

The specimens were studied using XRD to determine the influence of adding HPPM fibers to the concrete mixtures on the phase changes. Figure 3 shows the results of the 2% HPPM test at 28 d. The crystalline phases of portlandite Ca(OH)_2_, calcite Ca(CO)_3_, and silicon dioxide (SiO_2_) are the primary peaks. The Ca(CO)_3_ and Ca(OH)_2_ levels did not change appreciably when HPPM fibers were added. Similarly, when the fibers were added, they were observed at 650 intensity. This phenomenon demonstrates that the fibers are incapable of participating in chemical processes. Furthermore, the presence of amorphous materials is indicated by the convex form between 2 theta between 16° and 36°.

### Slump values

**Figure 4:**
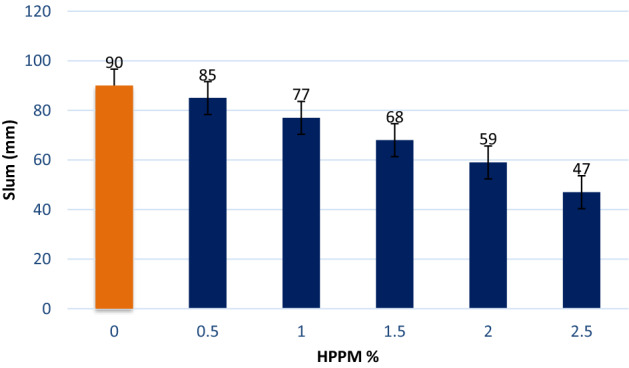
Slump values of different percentages of HPPM.

Figure 4 depicts the slump values of the concrete mixtures containing various amounts of HPPM as an additive. The slump values were expected to decrease linearly as the percentage of HPPM added to the concrete increased. In comparison to the reference slump of the reference sample, the slump values decreased by approximately 5%, 13%, 20%, 30%, and 43%, respectively. The decreased slump could be attributed to the HPP particle heterogeneity and roughness, which could diminish the fluidity of the mixtures as well as HPPM's high absorption (8.8%), as shown in Fig. 4. Because of the high porosity of HPPM (avg. 8.8%) and high cohesiveness between the HPPM and concrete matrix^29^, increasing the amount of HPPM resulted in lower slump values. The volume, form, and slenderness of the fibers, as well as the mix composition, influence the workability of concrete^1,9–11^. When the fiber dose exceeds this critical amount, the likelihood of fiber clamping or balling increases, resulting in an uneven fiber distribution and a greater reduction in flowability^27^.

### Uniaxial compressive strength (UCS)

**Figure 5:**
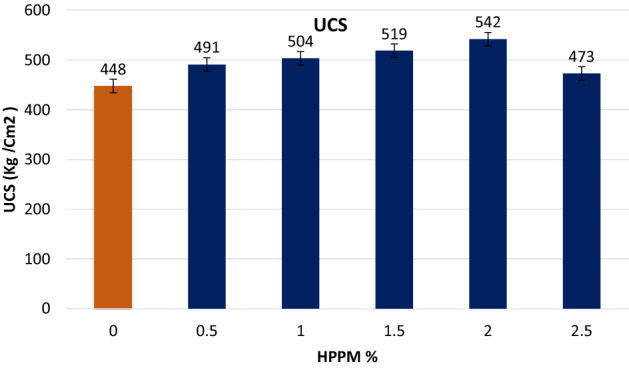
Compressive strength enhanced by the addition of HPPM after 28 days.

The UCS values of the samples are shown in Figure 5. The control mix in the experiment had a 28-d UCS of 448 kg/cm^2^, but the 2% addition of shredded HPPM by volume produced the best results. For the control mix study, UCS increased steadily between 1 and 1.5 % before falling marginally at 2.5 %. In comparison to the control sample, volume increments of 0.5, 1, 1.5, and 2% resulted in sample increases of 8.82, 11.05, 13.68, and 9.40 %, respectively (Fig. 5). The results showed that incorporating HPPM into concrete had a significant impact on the UCS of the mixture. In 2020, Xu et al.^7^ reported similar results for UCS, where the addition of different plastic fibers increased the UCS to the point where it began to fall. The improvement in UCS with the additional content of polypropylene fibers may be related to the fiber’s crack restriction effect, as demonstrated in earlier investigations^30^. According to a study by^31^, the declining trend at 2.5 % could be due to the presence of voids at 2.5 % and the existence of weakening interfacial connections between the cut-up HPPM and the cement (2017).

**Figure 6:**
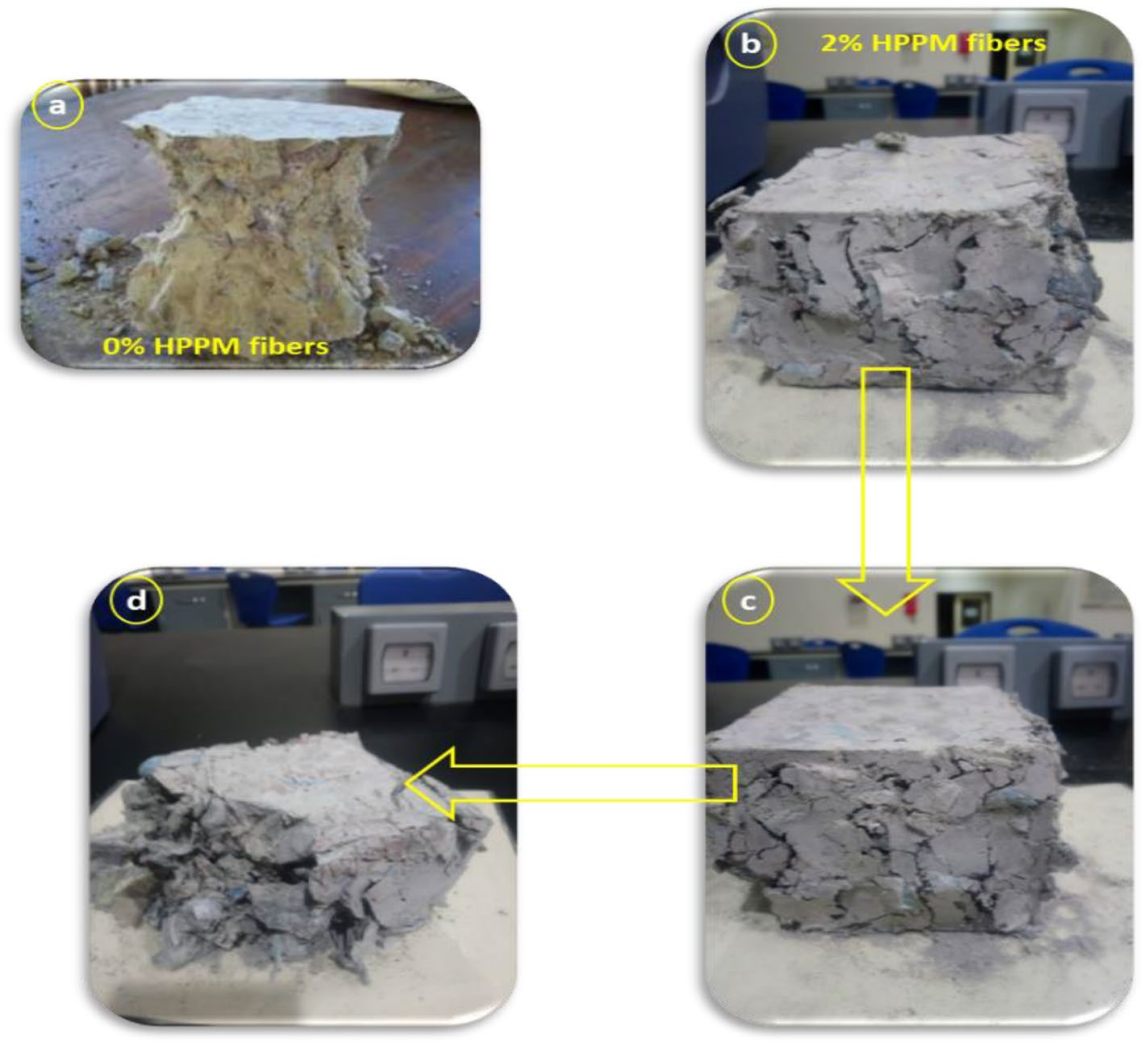
Failure mode of the concrete specimens without HPPM under (**a**) compression load (**b–d**), modifying the failure mode of concrete from brittle to ductile.

When the fiber dosage was increased from 0 to 3%, the UCS increased by 6%^32^. The addition of HPPM to the mixtures considerably modified the failure mode of concrete from brittle to ductile, as shown in Fig. 6. The specimens did not crush because of the bridging effect of the HPPM fibers, and they maintained their integrity until the completion of the test. It was discovered that mixtures containing HPPM had lower compressive strengths at an early age; however, after a longer curing period, they had higher compressive strengths. This suggests that the effect of bridging fibers can improve the UCS of concrete over time.

### Ultrasonic pulse velocity (Pv)

**Figure 7:**
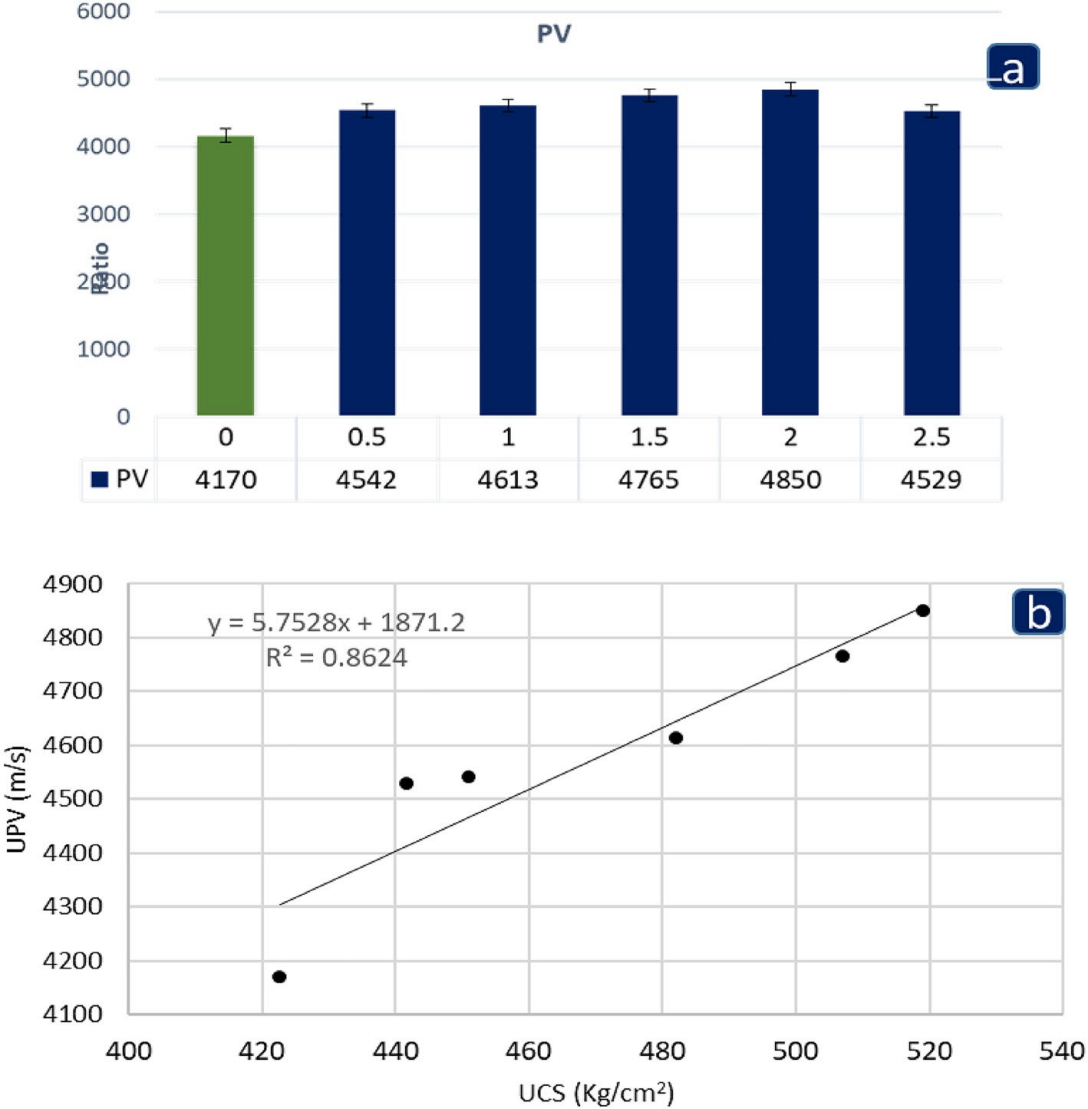
(**a**) P-wave velocity results after 28 days and (**b**) the relationship between UCS and PV for concrete containing HPPM.

At 28 d, an ultrasonic test was performed on water-saturated cubic concrete specimens of 150 mm to assess the number of internal pores in the specimens. This non-destructive test uses reflected waves that radiate between probes to assess the permeability of a specimen, according to^21^. The test was performed by crossing the two faces of the specimen with two probes of the instrument. The PV test is a nondestructive method for determining the consistency and efficiency of concrete. Concrete cracks and pores are also referred to as PV^33^. Non-destructive testing is a good way to assess the quality of concrete. The effects of the PV test are shown in Fig. 7a. PV grew consistently as HPPM content by volume increased until the volume crossed 2.0%, after which it declined marginally to 2.5%, as shown in Fig. 7a. Similarly, for UCS, the HPPM material with a volume of 2.0% produced the best results. According to^34,35^, concrete with a PV result of more than 4500 m/s is considered outstanding with a high-quality rating. The quality of the concrete decreased once again at the 2.5% volume mark compared to the control specimen in the experiment; regardless, the quality of the concrete improved in all mix designs, signifying beneficial features. According to^36^, good-quality concrete has no substantial voids or cracks in the ranges mentioned; consequently, as shown by the research of^37^, the usage of shredded facemasks reduced the number of microcracks in the concrete, thereby enhancing the overall quality of the concrete. The absence of voids or cracks can jeopardize the structural integrity of concrete within the above-mentioned limits. Owing to an increase in void content and thus porosity with increased fiber, PV values tend to drop beyond a fiber composition of 2.5%. According to BIS, the ultrasonic pulse velocity (UPV) values ranged between 3.8 and 4.04 km/s, indicating that the concrete quality is good^38^. The HPPM was added to the equation, which increased the UPV values up to a particular volume fraction. However, as expected, the increased HPPM stripe content resulted in lower UPV values. This decrease in the change in velocity is thought to be due to the existence of voids and microcracks in the concrete specimens, which reduce the homogeneity at higher fiber volume fractions. For specimens containing HPPM at any %, UPV values ranging from 4200 to 4600 m/s were discovered, and they were regarded as good quality concrete.

The UPV values are related to their matching cube compressive strength. The relationship between the UCS and UPV values of concrete mixtures that include HPPM fibers is strong, as shown in Fig. 7b. A power regression method was used to correlate the experimental results, with an R^2^ value of 0.872 for all samples, indicating a high level of confidence in the association.

### Sorptivity

**Figure 8:**
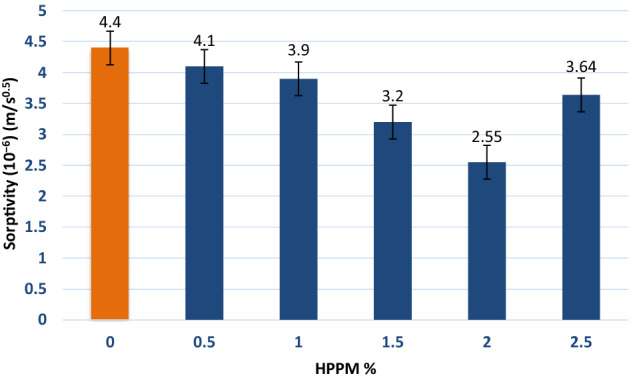
Sorptivity enhancement by adding HPPM after 28 days.

The sorptivity (S) results are shown in Fig. 8. When fiber concrete is compared to ordinary concrete, S, which is a measure of concrete durability, is lower. It is possible that the loss of connection in the pore space is caused by the porosity of the HPPM fiber filling porosity^39^. The minimum S for 2% HPPM is 2.55(106) m/s, whereas the maximum value for 2.5% HPPM is 3.46 m/s. Furthermore, all HPPM concretes had a lower S than the control mix, despite the fact that the high value for 2.5% HPPM was similar to a mix with considerable porosity. This result demonstrates the considerable reduction in the capillary n and inner conductivity of the pores when HPPM fibers are used, and it confirms all of the other durability findings in this study.

### Flexural strength

**Figure 9:**
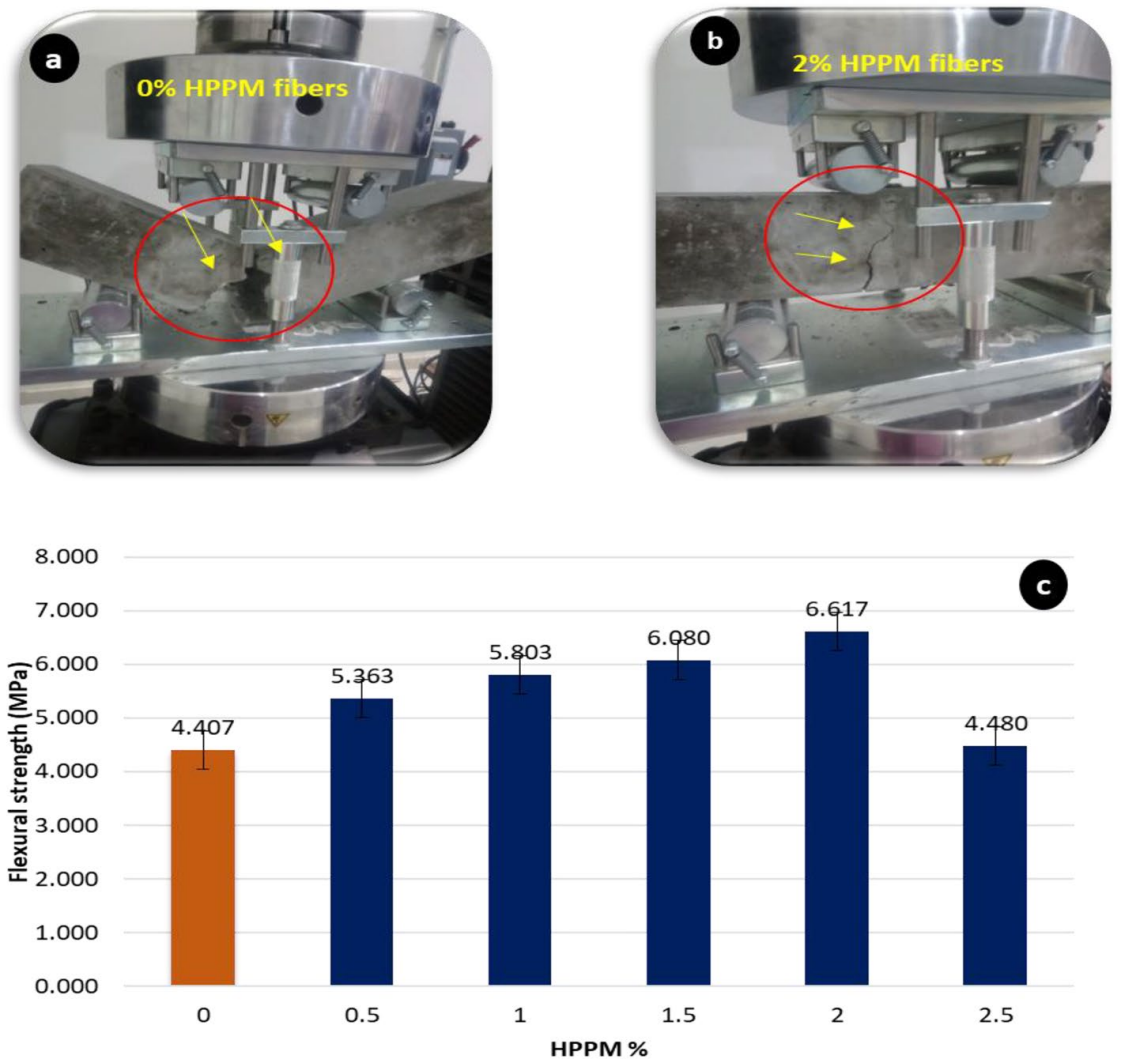
(**a**) Fibers intersecting the cracks in the tension zone of the specimens caused an increase in flexural strength (**b**) enhancement the FS after 28 days after adding the 2% HPPM (**c**) Influence of HPPM incorporation on flexural strength.

The FS of UCS and PV follows a similar pattern, with an increase up to 2% of HPPM fiber content and then a reduction as the number of fibers increases. The results shown in this graph (Fig. 9) indicate that, similar to the tensile strength, the concrete flexural strength increased as the HPPM concentration increased. In comparison to the control mix, specimens with HPPM of 0.5, 1, 1.5, 2, and 2.5% had FS of 17.8, 24, 27.5, 33.4, and 1.6%, respectively. Furthermore, HPPM plays a vital role in the development of FS, particularly after a longer water curing duration. The overall effect of HPPM appears to be geared toward increasing FS, as evidenced by the 33.4% increase in FS of concrete with 2.0% of HPPM. The decrease in FS as fiber content increases can be attributed to the fact that voids in the matrix grow as 2.5% HPPM fibers are added to the matrix. Upon applying HPPM to sustainable concrete, the FS of the sample was considerably boosted. As a result, further FS enhancements may be accomplished by introducing an HPPM with an optimized geometric shape to create a better concrete FS. The adoption of mechanically enhanced fibers with increased bond strength should result in a more resilient structural concrete capable of producing larger residual capacities as a result of developments in recycled HPPM processing.

The fibers intersecting the cracks in the tension zone of the specimens caused an increase in the FS. The HPPM fibers flex to hold the crack face separation, offering a larger energy absorption capacity and stress relaxing the micro-cracked area adjacent to the crack tip (Fig. 9a,b). This could be caused by the decreased workability of concrete at larger volume fractions in the mixtures. The recorded FS values of the prismatic beams are shown in Fig. 9c.

### Impact strength

**Figure 10:**
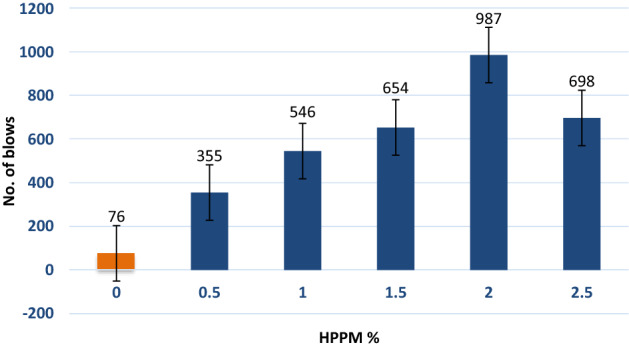
Influence of HPPM incorporation on Impact resistance.

Regarding the number of blows required to cause the concrete specimen to collapse, the IMs of concrete was investigated for different volume fractions of HPPM fibers. PPF improves the impact resistance of concrete^40^. The addition of just 1% micro PPF increased the number of blows till failure by nearly thrice^41^. The number of blows at first crack was assessed to be 76, 35, 546, 654, 987, and 698 percent for 0 percent, 0.5%, 1%, 1.5%, 2%, and 2.5% of HPPM, respectively, when HPPM was added to concrete mixtures. Furthermore, with 0%, 0.5%, 1%, 1.5%, 2%, and 2.5% of HPPM, the number of strikes required to destroy the sample increased 3.0, 3.3, and 4.8 times, respectively (Fig. 10). These findings are consistent with^42^, which found that the number of blows for failure increased from 76 (100%) for ordinary concrete to 355 (367.105%), 546 (618.421%), 654 (760.526%) 987(1198.68%), and 689 (818.421%) for concrete with HPPM fibers equal to 0%, 0.5%, 1%, 1.5%, 2%, and 2.5%, respectively, for concrete with HPPM fibers equal to mix control (Fig. 10).

### Spalling resistance

Furthermore, compared to concrete without fibers, the proportion of spalling for PPFRC is lower^43^. This is owing to advancements in fire protection. HPPM melts at 160 °C, whereas spalling occurs at 190 °C^44^. As a result, as fibers melt, empty channels emerge, and a new pathway is generated for gas to escape. Simultaneously, it lowers the internal pore pressure. These findings were also confirmed by^45,46^ and others. Finally, the HPPM considerably improved the fire resistance of the concrete.

### Abrasion resistance

The use of HPPM fibers improves the abrasion resistance of concrete. Horszczaruk^47^ demonstrated that after including 0.9 kg/m^3^ of fibers, the mean depth of wear for HPPM fell from 29 to 42% compared to that of plain concrete. The increase in abrasion resistance of concretes containing fibrillated 0, 0.5, 1, 1.5, 2, and 2.5% HPPM varied from 6.4, 5.7, 4.9, 3.7, to 4.6%, depending on the water to cement ratio^48^.

**Figure 11:**
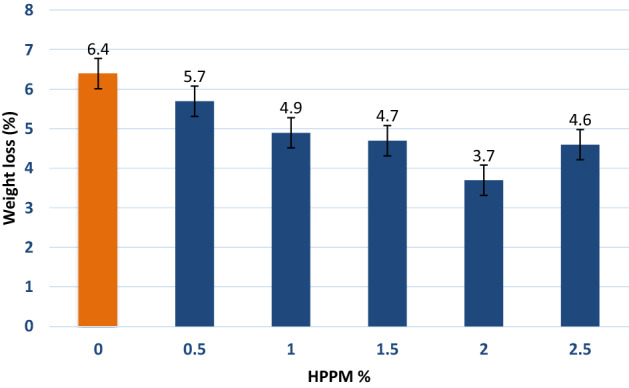
Abrasion resistance enhancement 28 days after adding the HPPM.

This phenomenon can be explained by the fact that incorporating HPPM fibers into concrete inhibits the creation of cracks and effectively diminishes its intrinsic cracking tendency. Furthermore, the pore-blocking effect of HPPM fibers causes the pore structures in hardened concrete to become more detached, resulting in less capillary porosity and lower water penetration of the concrete. In addition, HPPM’s abrasion resistance strength improved. Regarding the resistance to abrasion damage, the HPPM fibers outperformed the control concrete (Fig. 11).

### Water absorption and porosity

The resistance of concrete to the intrusion of hostile ions is another important factor that influences its durability. The porosity of concrete is indirectly represented by its absorption characteristics, which provide useful information regarding the permeable pore volume within the concrete and the connectivity between these pores^49^. The percentage of Sw is a measure of the pore volume or n of concrete after hardening, and it is one of the fundamental factors of concrete durability.

Regarding water absorption, many studies have shown that HPPM absorbs less water than plain concrete. According to^50^, regular concrete absorbs 1.52% water, whereas concrete containing 1.5, 3.0, or 4.5% PPF absorbs 39, 46, or 49 % of water, respectively. Similarly, in^51^ the water absorption was reduced by approximately 45%, from 2.481 to 1.366%. PPFRC absorbed 24.7 % less water than concrete without fibers in prior tests^52^. This could be due to the action of the fibers, limiting the number of cracks to a minimum. Fibers, however, have been shown in some tests to have a negative impact on absorbability.

**Figure 12:**
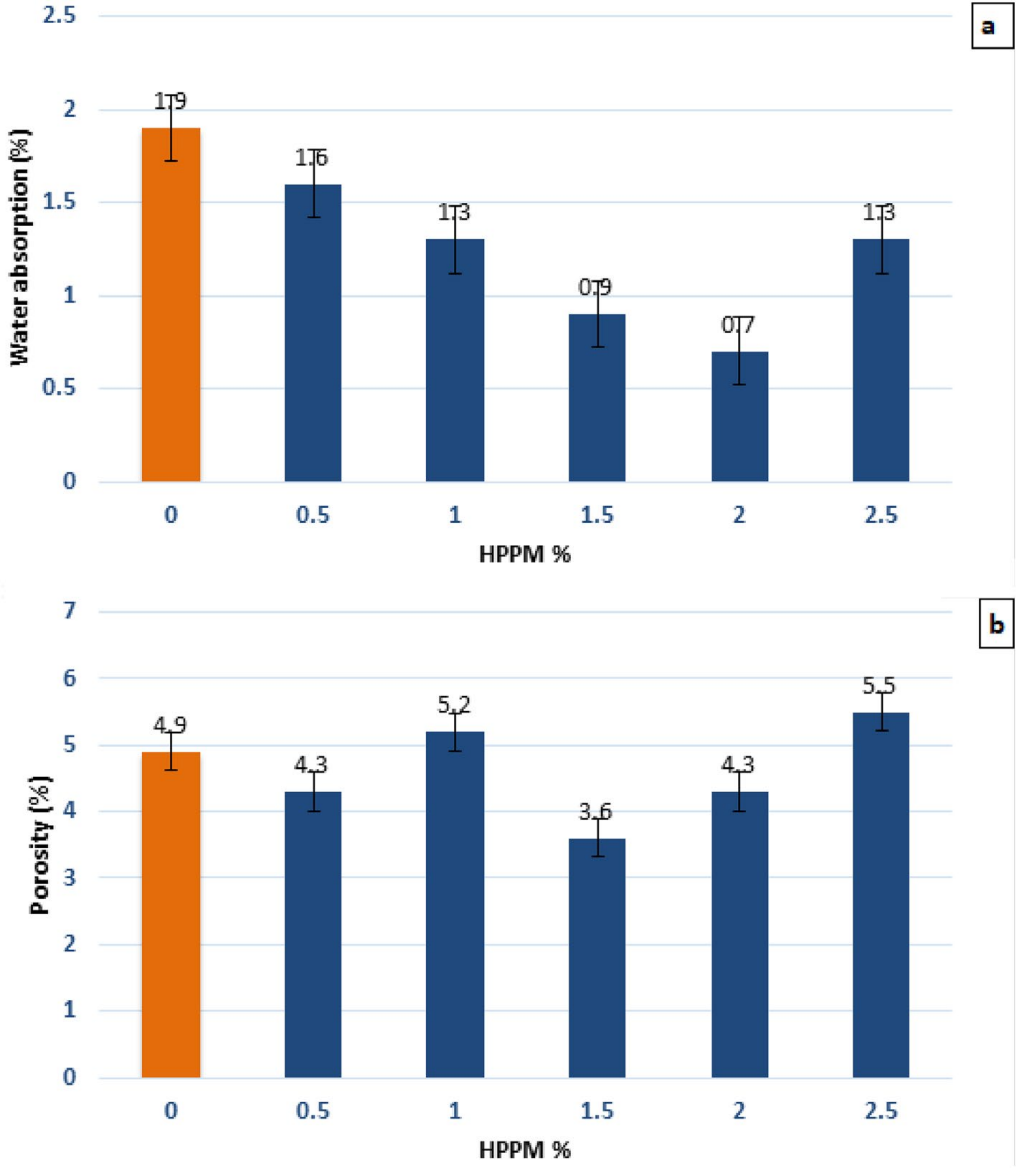
Influence of HPPM on (**a**) water absorption (Sw) and (**b**) porosity (n) as reported in selected studies.

Our findings show that injecting HPPM into concrete significantly reduces its Sw. When compared to the respective values obtained from control concrete mixtures, the water absorption of concrete mixtures with 0, 0.5, 1, 1.5, 2, and 2.5% HPPM decreased by 25% and 36%, respectively. The results of polypropylene HPPM fiber-reinforced concrete show that HPPM fibers favorably reduce the concrete water absorption. As shown in Fig. 12a, the increased fiber content resulted in a greater reduction in water absorption. As a result, among all HPPM fiber-reinforced concretes evaluated in this study, the combination containing 2% HPPM fiber exhibited the lowest water absorption. Adding fibers to concrete provides a variety of advantages, but it also causes the thickness of the transition zone to increase in hybrid fiber-reinforced concretes.

The influence of HPPM on porosity cannot be clearly measured, as shown in Fig. 12b. Workability is influenced by a number of factors, one of which is the distribution of fibers within the mixture and the level of porosity. Studies have shown that when the fiber dosage increases, the porosity increases^53^. Porosity can decrease when the addition of fibers is confined to a lower amount, and then it can increase again with larger fiber additions, as shown previously^54^. The porosities of the concrete with 0, 0.5, 1, 1.5, 2, and 2.5% HPPM were 4.9, 4.3, 5.2, 3.6, 4.3, and 5.5%, respectively, in this study. A summary of the effect of HPPM fiber incorporation on concrete porosity is provided in Fig. 12b. Adding more than 2.5% of HPPM fibers to the concrete resulted in increased transition zone thickness and n and thus a higher Sw. The increased porosity could be due to poor compaction, which could lead to more micro-cracks, unrestrained fibers, cracks, and poor fiber–matrix bonding^55^.

### Water penetration and permeability

Because of the pore blocking action of HPPM fibers, all water penetration depths for HPPM were lower than those for the control mix. These findings support the accuracy of the HPPM results.

**Figure 13:**
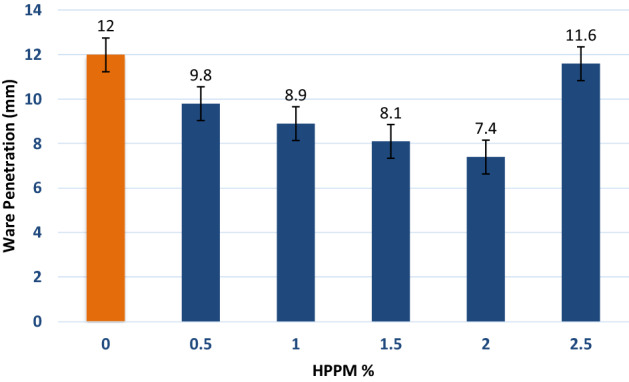
Depth of water penetration versus HPPM fiber content after 28 days.

The specimen with 2% HPPM fiber content had a minimum depth of penetration of 7.4 mm, which was 38.33% less than that of the control mix. The decrease in HPPM depth of water penetration and then increase (11.6 at 2.5%) could be attributed to an increase in “n” as the HPPM fiber content increased. In fact, the 2% decrease in water depth is most likely attributable to pore blockage and decreased capillary porosity. This result supports the results of other durability tests presented in this study. Figure 13 shows the depth of water penetration at 28 d.

In addition, the effect of HPPM fibers on permeability is not well understood. ^3^ found that adding PPF to concrete increased both water and gas permeability. Hager et al.^56^ reported a similar finding. Many studies, on the other hand, have discovered that fibers have a favorable effect on permeability. According to^57^, the addition of PPF to concrete reduces the duration of water permeability. Similarly, samples with fibers demonstrated poorer permeability than samples without fibers^58^. Studies have also shown that permeability declines as the volume of fibers increases up to a certain point, then increases, and sometimes exceeds that of plain concrete^59^. This is usually due to a lack of workability and an excessive amount of fibers in the mixture.

The presence of HPPM fibers in concrete reduces the likelihood of the concrete breaking by limiting crack formation. Fibers also cause the pore structures in hardened concrete to become more separated, resulting in a lower capillary porosity and concrete water penetration.

## Role of stripes in pore blocking and strength improvements

### Microstructure analysis

**Figure 14:**
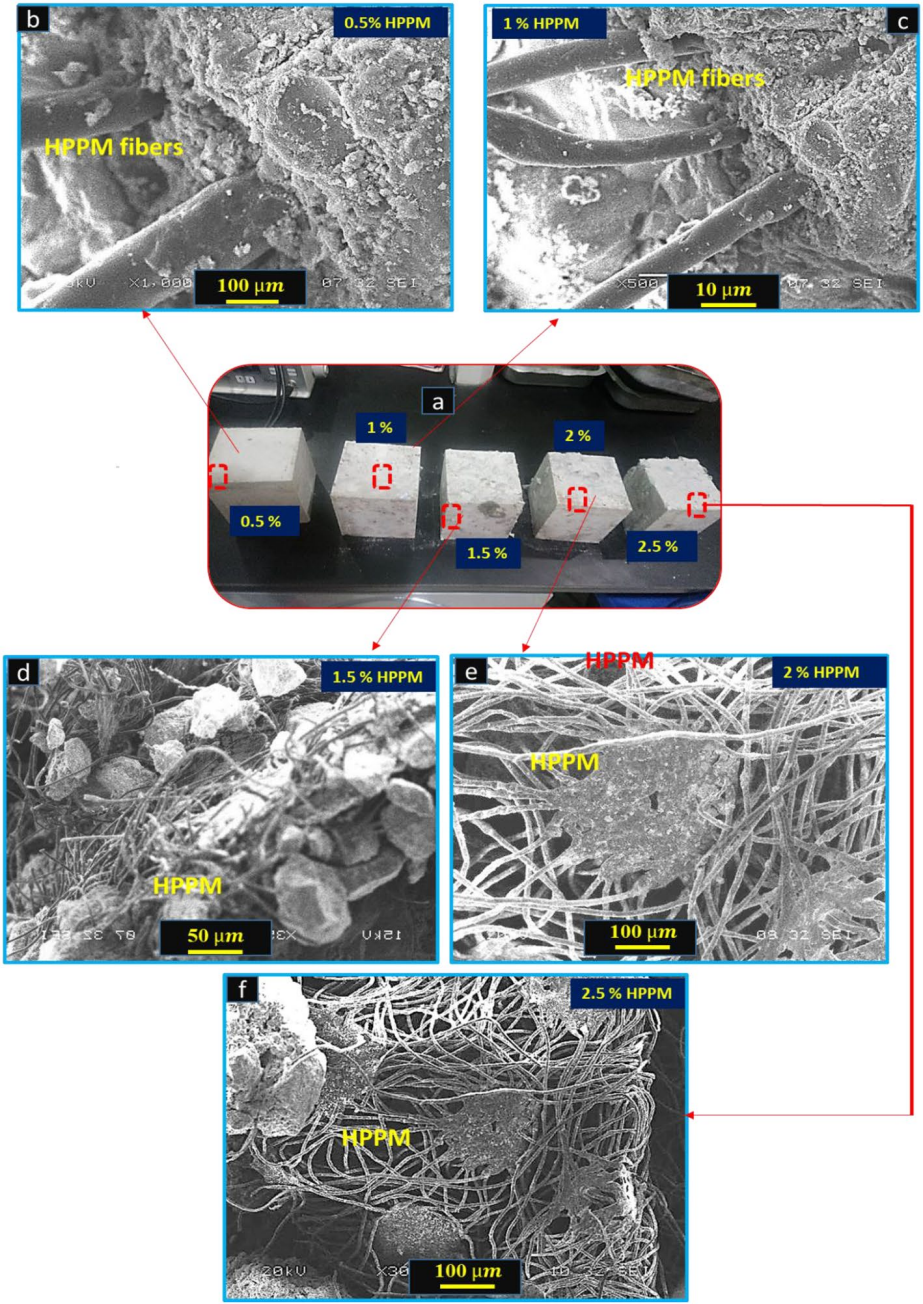
SEM images of concrete with HPPM fibers. (**a**) Cubes with different percentages of HPPM, (**b**–**f**) 0.5, 1, 1.5, 2 and 2.5% of HPPM fibers.

The microstructure of the HPPM fibers with 0.5, 1, 1.5, 2, and 2.5% volume fractions were examined using SEM to evaluate the bond characteristics of the mixture. Figure 14a,b depict the HPPM fiber–matrix interface of a concrete composite that included HPPM fibers and fiber bridging following a fracture. The results of compressive and flexural tests on the concrete incorporated with HPPM fibers showed that the fiber–matrix interface was stronger and that the fiber–matrix bond was stronger.

Another disadvantage of concrete is that it breaks almost immediately after it is poured and before it is fully set. These fissures are a key source of concrete weakness, especially in large-scale worksite applications, causing fracture and failure, as well as a general lack of resilience^60^. Traditional reinforcement and, to a lesser extent, the use of a suitable amount of particular fibers will assist in overcoming tension weakness^61^. The microstructure of HPPM concrete with a 2% volume fraction of HPPM was investigated using SEM to assess the bond characteristics of HPPM in the mixture. The microstructures at 0.5, 1, and 1.5% of the HPPM surface and hydrated cement matrix after the fracture of the concrete specimen are shown in Fig. 14b–d, respectively. The surface of the HPPM was coated with a densely hydrated cement matrix, as shown in Fig. 14b–d. This event shows that the HPPM and the wet cement matrix formed a strong connection.

The HPPM and cement matrix had a strong interfacial connection, as shown in Fig. 14e. This bonding was important for reducing the size and number of cracks, which led to a 2% increase in HPPM strength. The bridging activity of the fibers, upon which the bridging fibers partially transferred the stress across the crack, could also account for the improved flexural performance of concrete containing HPPM. Similar findings were reported by^30^, who discovered that adding polypropylene to concrete substantially increased its flexural strength.

At 2% HPPM fiber, the highest compressive strength values were attained. The highest UCS increases measured at 1.5% and 2% HPPM were 13.6% and 9.40%, respectively. Therefore, it is reasonable to conclude that stripes have a significant effect on the compressive strength. According to these findings, the HPPM stripes have a considerable effect on the UCS values compared to those of the control concrete. The high fineness and variable length of fibers in staple HPPM stripes form a network that works as a bridge, preventing the microfracture from spreading further. When the HPPM stripe level was higher (2.5%), however, the fiber stripes were dispersed unevenly in the concrete owing to poor workability and mixing. As a result, these fiber masses collected to generate weaker locations (Fig. 14f).

After the flexure test, the fibers operated as a bridging element, effectively transferring the load from the matrix to the HPPM fibers, allowing them to take on the additional load, and resulting in an increase in the UCS and FS compared to those of the control concrete. The size and shape of the polypropylene fibers affected the increase in the flexural strength of the concrete. Additionally, as a result of the lower effective w/c ratio, the splitting tensile and FS values were relatively high, with 17.8, 24, 27.5, 33.4, and 1.6% increases in FS at 0.5, 1, 1.5, 2, and 2.5% HPPM, respectively, compared to those of control samples. In a scattered striped cement matrix, the stress concentrations are not uniform along the length of the fiber.

The combination of HPPM stripes and fibers is a factor that improves the FS. The HPPM stripes flexed to keep the fracture face apart, offering a larger energy absorption capacity while relaxing the microcracked area adjacent to the crack tip. However, a higher fiber content (2.5% HPPM) resulted in a reduction in FS (Fig. 14f). This could be caused by the decreased workability of concrete at larger volume fractions in the mixtures. Inadequate compaction, more micro-cracks, uncontrolled fibers and cracks, and poor fiber–matrix bonding could all contribute to the increased permeability and porosity. HPPM acts as a three-dimensional reinforcement, bridging cracks, and preventing growth and enlargement^62^. Importantly, cracks are not detrimental to construction or serviceability if they do not exceed a particular size. When concrete transitions from a plastic to a solid state and the Young’s modulus of the concrete exceeds the Young’s modulus of the fibers, micro HPPMs are no longer considered to play a significant role. Furthermore, in a prior study^63^, the cracking area in concrete with 0.5% PPF was reduced by 99%. PPF prevents cracks from forming not only against plastic shrinkage but also against drying shrinkage^11,63^.

However, as shown in Fig. 14f, 2.5% HPPM had a higher porosity, causing the specimen to be non-homogeneous. Furthermore, the HPPM fibers have a bridging effect, which can lead to increased compressive and flexural strengths. In reality, lower permeability and capillary porosity are closely related. It may be deduced that the voids in 2.5% HPPM, which are more than those in 2% HPPM, are caused by HPPM fibers trapping air in the mixture. Furthermore, the HPPM stripes and fibers can act as a binder all over the fibers and aggregated in this micrograph, potentially causing pore blockage and decreased permeability. In reality, lower permeability and capillary porosity are closely related. The voids in 2.5% HPPM, which are more than those in 2% HPPM, are likely caused by fibers that have trapped air in the mixture.

### Geometry and crack-strengthening mechanisms

Some researchers have recently emphasized the effect of fissures on concrete permeability^64^ investigated the effect of the fracture width on concrete permeability in an experimental setting. Shin et al.^65^ studied the influence of concrete permeability on fracture type, crack width, and water head. Yang et al.^66^ used X-ray CT to monitor the water transport parameters of cracked concrete and indicated that fracture morphology and tortuosity should be studied in future research.

**Figure 15:**
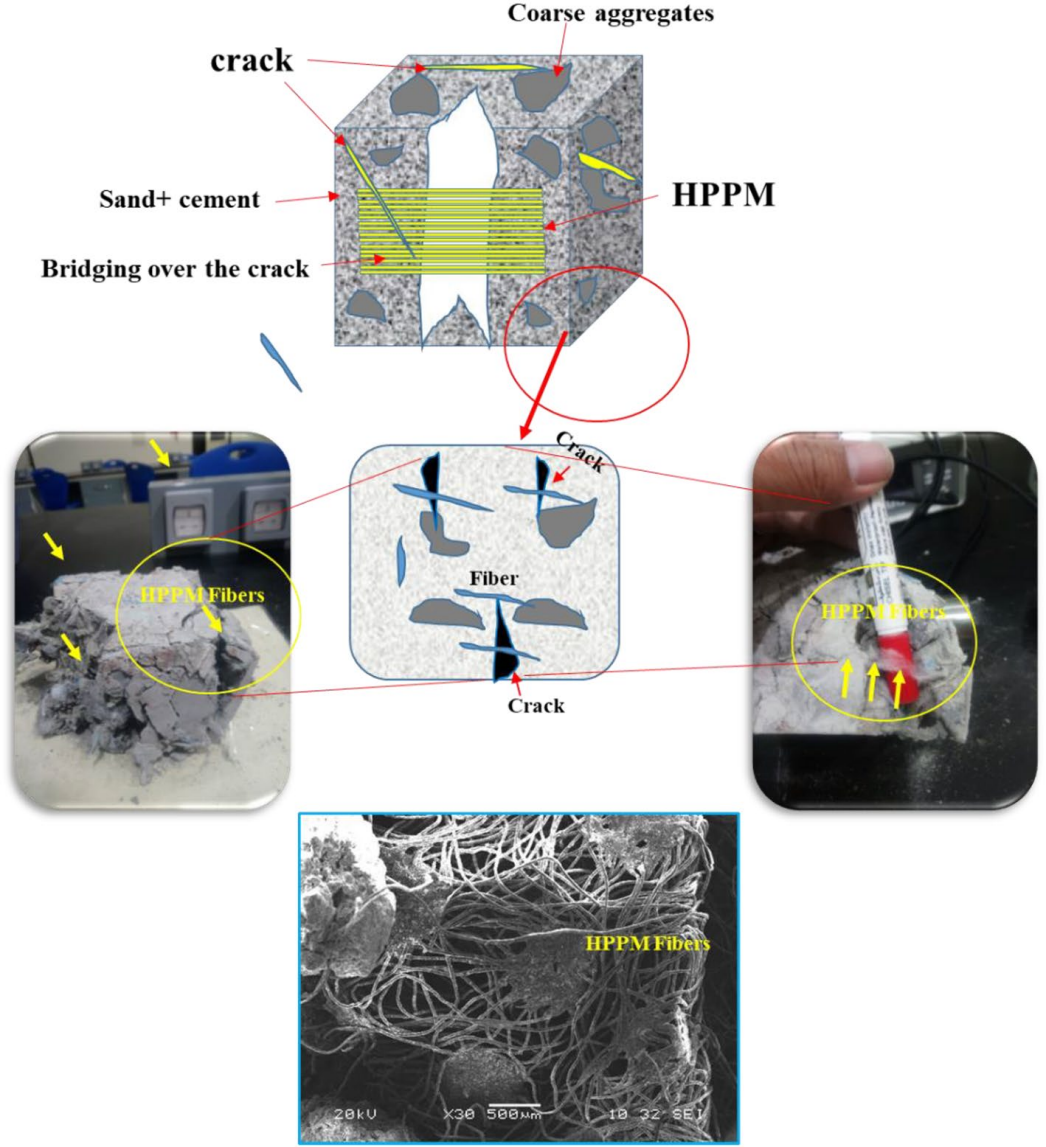
Schematic representation of the mechanism of bridging formed by HPPM at 2% fibers which they acting as. Crack-bridging activity during the failure.

The main role of HPPM in concrete construction is shown in Fig. 15. As shown, the stresses arising from plastic shrinkage exceed the concrete strength in the initial hours of its age, when both the strength and Young's modulus are quite low. As a result, shrinkage cracks form. The formation of cracks is slowed by a high number of equally dispersed HPPM, which reduce the crack width by two orders of magnitude^5^. Of note, cracks are not detrimental to construction or serviceability if they do not exceed a particular size. When concrete transitions from a plastic to a solid state and the Young’s modulus of the concrete exceeds the Young's modulus of the fibers, micro HPPMs no longer play a significant role. In this study, concrete samples with no fibers had a breaking area of 1743 mm^2^ in the study^67^, whereas those containing 0.5 and 1.0% PPF had cracking areas of 992 and 99 mm^2^, respectively. According to this study, the presence of HPPM increases the drying shrinkage resistance of concrete.

The addition of macro HPPM fibers can change the fracture shape based on the aforementioned physical and mechanical data. This behavior can be attributed to the macro polypropylene fibers, which are scattered stochastically in the matrix, preventing the matrix fracture from propagating without stretching and debonding the fibers, resulting in a deviated crack-extension route. When comparing HPPM fiber specimens to control concrete samples, specimens with a fiber dosage of 2.5% showed the most significant increase in crack morphology.

The use of fibers from various raw materials is efficient in controlling crack formation on exposed concrete surfaces caused by early-age drying shrinkage^37^. Because they restricted the motions of the micro-level in concrete by bridging and stitching fine fractures, the PPF in concrete decreases the drying shrinkage and early cracking^5^. The effect of HPPM on matrix fracture behavior can be divided into two categories. First, the addition of HPPM fibers reduces the maximum stress as well as the elastic modulus of the mixtures. Second, after the grain-bridging crack face breaks, the stress can be transferred via cracks through the intersection of the fibers and cracks. Crack-bridging activity results in increased ductility in HPPM-fiber-reinforced concrete (Fig. 15).

### Eco-friendliness and economic feasibility of HPPM

In today's construction business, the issues of susceptibility and environmentally friendly materials are hotly debated^12–14,68,69^. CO_2_ concentrations in the environment have increased by 50% in the twenty-first century^2^. Concrete manufacturing accounts for 2–3% of annual energy demand and 8–9% of total CO_2_ emissions in the atmosphere^70^. Consequently, the construction industry now faces a new challenge: manufacturing concrete structures that meet environmental standards while also being more durable. Protecting steel bars from corrosion and sulfate attack improves the longevity of reinforced concrete, allowing water and ions to penetrate through cracks and pores^2^. Plastic shrinkage at an early age is widely recognized as one of the leading causes of concrete cracks. As a result, the concept of HPPM fiber inclusion appears to be quite useful in terms of long-term development. Ali et al.^71^ conducted a comparative study of plain concrete and concrete containing various types of fibers, including steel, glass, and PP. It was discovered that the manufacturing of PPF produced 30 and 9% less CO_2_ than steel and glass fibers, respectively. The environmental and economic issues associated with pavements made from the various concretes described above were evaluated. In addition, depending on the fiber dose, carbon emissions per m^2^ of pavement were reduced by 13–18%. The thickness of concrete pavement was reduced by 18% in another study^72^ owing to the use of PPF. The COVID-19 pandemic caused a global crisis with social, economic and environmental consequences^73^. Inappropriate management of spent HPPM is another plausible pathway for COVID-19 transmission. The present work encourages scientists to express their concerns to governments at all levels regarding the significance of implementing suitable solid waste management measures, such as HPPM, to prevent the spread of the novel coronavirus. With 50% of its population, Saudi Arabia is the most populous country in the Arabian Peninsula. To date, there have been 417,363 coronavirus cases and 6957 deaths. A total of $375,831 has been recovered (www.worldometers.info). As previously stated, the primary application of fiber-reinforced concrete is in the construction of structural elements. The prospect of employing such concrete to build architectural shapes is rarely considered. Public spaces are one of the fields of application in architecture. Public open spaces are one of the most important aspects of city life^74^, and their attractiveness affects how people view the city. Furthermore, as a result, cities are viewed as pleasant and inviting to people. HPPM stripes can be used to produce architectural shapes in public spaces, such as decorative pavements in scientific centers, shopping centers, restrooms, promenades, zoos and gardens, bus stations, parking areas, ferry terminals, feck rocks, beach areas, landscapes, door surrounds, and skate parks. Finally, characteristics of the materials utilized in these spaces should be considered.

### Specific methods to minimize the HPPM potential for risk

Various efficient methods for cleaning the gathered HPPM are currently being used to convince the construction industry to use masks and other PPE waste without the risk of disease transmission. These techniques are as follows:

1. To help with supply constraints caused by the COVID-19 pandemic, the FDA in the United States has approved hydrogen peroxide in vapour (VH_2_O_2_) for high-throughput decontamination of PPE, either alone or in combination with ozone.
2. UVC decontamination techniques, in particular those that utilize this wavelength, can also be used as a potential HPPM decontamination method. Other UV technologies are also used to avoid process harmonization.
3. Moist heating at 60–70 °C for 60 min in conjunction with high humidity is a viable decontaminating technology for HPPM since it offers scalability and high-throughput processing. For decontaminating PPE, microwave-generated steam would be ineffectual.
4. Physical irradiation methods such as ethylene oxide, electron beams, and gamma radiation are useless for recycling HPPM^75^.

## Limitations and future work

Materials utilized in earlier studies have varied in size and length/width ratio. Kilmartin-Lynch et al.^12^ have employed tiny pieces that measure 2 cm in length and 0.5 cm in width. Koniorczyk et al.^13^ used samples that were 0.5 cm long and 0.4 cm wide, whereas Ran et al.^15^ used samples that were 2 cm long and 0.4 cm wide. In this article, the HPPM was cut into small pieces with a length of 1 cm and a width of 1 cm. The scope of this work does not include size effect and the length/width ratio effect, which need further investigation in future work.

## Conclusions

The main function of HPPM stripes in concrete construction is to reduce plastic shrinkage cracks. Changes in the mixture can improve many characteristics of HPPM-fiber-reinforced concrete. However, certain qualities have insignificant effects or are difficult to assess. Of note, the conclusion that a higher HPPM fiber content results in better characteristics is not always correct, and an excessive amount of fibers can cause substantial deterioration. For example, polypropylene fibers improve the material characteristics up to a specific dosage, which, if exceeded, has detrimental consequences. When determining the ideal HPPM fiber content, it is critical to consider the mixture composition and fiber quality. HPPM fiber-reinforced concrete applications in open public places are a promising field. Because concrete is subjected to adverse environmental conditions, damage, abrasion of the surface, and vandalism, using concrete with enhanced quality is definitely advantageous.

In sustainable concrete, various percentages of iron waste (0, 0.5, 1, 1.5, 2.0, and 2.5%) were employed. The slump, compressive strength, flexural strength, abrasion resistance, impact resistance, sorptivity, spalling test, water absorption, porosity, water penetration, permeability, and UPV were investigated. The following conclusions were drawn based on the results of these experiments.

- When HPPM fibers are added to the concrete mixture, they reduce the workability of the new concrete and have the potential to improve some of the mechanical qualities of the concrete when utilized in small amounts.
- Compared with the control mix, HPPM improved the overall quality of the concrete because the fibers were more evenly spread, resulting in higher compressive and flexural strengths. Because it restricted the movements at the micro-level in concrete by bridging and sewing fine cracks, HPPM decreased the drying shrinkage and early cracking in the concrete. The mixture with 2% HPPM fiber content yielded the highest compressive and flexural strengths.
- XRD showed that after introducing PP fibers, neither Ca(CO)_3_ nor Ca(OH)_2_ was altered appreciably. This demonstrates that the fibers are incapable of participating in chemical reactions. The presence of fibers causes a considerable change in the cracking pattern of the concrete. While unreinforced concrete forms wide and long cracks, the inclusion of fibers reduces the crack width opening, cracked area, and crack propagation through the bridging activity of the fibers. Crack networks become less connected as a result of adding the fibers.
- Water absorption in hardened concrete is reduced when HPPM stripes and fibers were added separately in low-volume fractions to the concrete mixture. Furthermore, the use of HPPM fibers in small volume fractions reduced the porosity of the concrete specimens. Among all the concrete specimens, the concrete containing 2% HPPM fibers had the lowest water absorption and porosity percentage. Regardless of the volume concentration of HPPM, all concrete mixes were subject to the same high standard of excellent quality and structural strength.
- Microfractures propagate along concrete with HPPM, according to a concrete microstructure study. As demonstrated in SEM images, HPPM fibers (2%) play a significant role in fracture bridging. However, when the volume percentage of the fibers is higher (2.5% of HPPM), voids form and grow between the cement paste and the fiber, reducing the concrete's strength qualities. As a result, HPPM can be utilized to make sustainable concrete and produce a clean, environmentally friendly building material. In addition, the HPPM fibers employed in this study were classified as short or discontinuous, which could lead to increased UCS, FS, and abrasion resistance.

## Data Availability

The datasets used and/or analysed during the current study available from the corresponding author on reasonable request.

## Abbreviations

PPs: Polypropylene stripes
HPPM: Healthy personal protective materials
FM: Single-used face mask
UCS: Uniaxial compressive strength
FS: Flexural strength
PV: Ultrasonic pulse velocity
SEM: Scanning electron microscope
XRD: X-ray diffraction patterns
Ηe: Porosity
Sw: Water absorption
IMs: Impact strength
AbR: Abrasion resistance
S: Sorptivity coefficients
